# Controversies in the Management of Portal Vein Thrombosis in Liver Cirrhosis

**DOI:** 10.3390/jcm9123916

**Published:** 2020-12-02

**Authors:** Andrea Mancuso

**Affiliations:** Medicina Interna 1, ARNAS Civico—Di Cristina—Benfratelli, Piazza Leotta 4, 90100 Palermo, Italy; mancandrea@libero.it; Tel.: +39-3298997893; Fax: +39-0916090252

## 1. Introduction

Portal vein thrombosis (PVT) is frequently diagnosed in advanced-stage liver cirrhosis, with a reported prevalence of 10 to 25% [1,2]. However, whether PVT directly affects the outcome of cirrhosis is controversial. In fact, although PVT corresponds to a poor prognosis in patients with cirrhosis, whether it is a cause or a consequence of liver function derangement remains unknown [3]. Consequently, the benefits of PVT treatment in cirrhosis are a continued subject of debate [1,2,3].

Cirrhosis is not only predisposed to bleeding but also constitutes a prothrombotic condition [1]. Moreover, it has been speculated that micro-vascular ischemia contributes to fibrogenesis in cirrhosis [4,5,6,7]. In clinical practice, fear of an increased risk of complications often causes patients with cirrhosis to opt against treating PVT. The aim of this paper is to report an update on the controversial issue of PVT management in cirrhosis and on the outcome of not treating it.

## 2. Does PVT Worsen the Outcome of Cirrhosis?

A meta-analysis from the United States [8] aimed to assess the influence of PVT on both mortality and hepatic decompensation in cirrhosis; it included only three studies and 3735 patients with cirrhosis, 260 of whom had PVT, after a strict selection process [9,10,11]. The study concluded that PVT significantly affected both mortality and hepatic decompensation [8]. However, this meta-analysis should be evaluated with caution, as it included studies with heterogeneous populations (partial and branch PVT cases were excluded in one study, and partial and total PVT cases were mixed in another). Moreover, the results of the meta-analysis [8] contradict those of many other (primarily retrospective) studies, which have predominantly reported that PVT had no impact on the mortality of patients on waiting lists for liver transplant (LT) [12,13,14,15,16,17,18].

Furthermore, a prospective multicenter study, in which 1243 patients with cirrhosis were screened with an abdominal ultrasound to reveal 118 new cases of PVT (the largest cohort of patients with PVT in cirrhosis currently published), reported that PVT was not a prognostic factor for mortality or hepatic decompensation [19].

In a recent monothematic issue on splanchnic vein thrombosis [20], another group published a study on the association between PVT and survival in cirrhosis. Thirteen selected studies were systematically reviewed. However, a meta-analysis was not conducted because of significant heterogeneity in data reporting and the lengths of follow-ups across the studies [21].

A long-term multicenter study included 604 patients with splanchnic vein thrombosis with and without cirrhosis. The authors compared splanchnic vein thrombosis and cirrhosis with nonmalignant noncirrhotic splanchnic vein thrombosis and found that the incidences of both major bleeding episodes and thromboembolic events were significantly higher in the former [22].

An additional study on the natural course of extrahepatic nonmalignant partial portal vein thrombosis in 42 consecutive patients with cirrhosis and extrahepatic nonmalignant untreated partial PVT found no association between the progression or regression of partial PVT and clinical outcomes (2-year survival, decompensation, hospitalization, need for LT) [23].

Recently, a meta-analysis of 33 studies, which included 1696 cirrhotics, reported that anticoagulation (AC) therapy significantly improved overall survival without influencing overall bleeding [24]. However, the surveyed studies included heterogeneous populations and were predominantly retrospective; some were presented only in an abstract form, and none were randomized or controlled. In the absence of randomization, combining data from cohorts in which the primary option was treatment with those in which it was no treatment exacerbates the risk of bias in the selection of patients [24].

## 3. Efficacy and Risks of Treatment for PVT in Cirrhosis

The risk of anticoagulant-related adverse events in patients with PVT and cirrhosis has been under-studied. A systematic review and meta-analysis provided strong evidence that anticoagulation therapy is associated with increased rates of recanalization and reduced progression of thrombosis, compared with no anticoagulation treatment. However, this systematic review was based on data on anticoagulant-related bleeding in only 257 patients [25], which is an insufficient sample size for an adequate evaluation. Indeed, if the benefit-to-risk ratio of anticoagulation therapy in other contexts, such as atrial fibrillation, was evaluated in trials that included several thousand patients, the results differed [26,27,28,29,30,31,32,33,34,35]. Similar results and limitations were recently reported in a systematic review with a meta-analysis from China [24]. The above data cast doubt on the negative role of PVT in the natural history of cirrhosis. Furthermore, the real risk-to-benefit ratio for treatment is difficult to evaluate.

Several studies have focused on anticoagulant treatment or TIPS [1,2,3]. The recommendation in the EASL Clinical Practical Guidelines on vascular diseases of the liver is to consider anticoagulant administration at a therapeutic dose for at least 6 months, provided with adequate prophylaxis for gastrointestinal bleeding, as a treatment for PVT in cirrhosis. Moreover, once PVT has been re-permeated, the guidelines suggest prolonging anticoagulation therapy for several months or until transplant in liver transplant candidates. Furthermore, in liver transplant candidates who have progressive PVT that does not respond to anticoagulation treatment, patient referral for TIPS should be considered [1]. However, these recommendations may need further confirmation because of uncertainties in the prognostic role of portal thrombosis in the natural history of cirrhosis. In fact, several issues involving treatments for PVT in cirrhosis remain to be elucidated. One unresolved issue is whether such treatments prolong survival or, more specifically, which treatment subgroups are expected to be associated with ameliorated outcomes. Currently, there are no data that compare AC and TIPS as treatments for PVT in cirrhosis. Finally, when approaching patients with PVT and cirrhosis, the treatment decisions become markedly more stringent if the goal is to facilitate LT. However, no well-designed controlled studies are yet available on this topic [26,27,28,29,30,31,32,33,34,35].

## 4. Suggestions for Preventing PVT in Cirrhosis

As the role of PVT in the natural history of cirrhosis is controversial, one would expect to see interventions fail to prevent PVT in cirrhosis. Instead, a single-center randomized controlled trial to evaluate the safety and efficacy of enoxaparin (Ex) in preventing PVT in 70 patients with advanced cirrhosis reported a significantly reduced PVT incidence, lower liver decompensation, and better survival in the treated group [36]. However, in a recent large multicenter prospective study, the rate of PVT in the untreated control group (27.7% incidence of PVT in controls at 96 weeks) was significantly higher than 10% at 5 years [19]. It is true that, in the latter study, cirrhosis was less advanced (Child–Pugh classes A and B), raising concerns about the impact of the severity of cirrhosis on PVT occurrence, and it is not possible to exclude a selection bias due to the small sample size in the former study [36]. Even if the results of this study are accepted [36], it is also possible that the effect of AC was not related to the prevention of PVT but to a beneficial impact on liver cirrhosis; a potential reason that treatment with enoxaparin resulted in a significant improvement in survival might be associated with the theory that cirrhosis development can arise from chronic micro-vascular ischemia, the so-called ischemic liver cirrhosis (ILC) theory. In fact, chronic liver injury might result in endothelial damage and micro-vascular thrombosis, triggering inappropriate hepatocyte proliferation and fibrosis [4,7].

## 5. Data about Untreated PVT in Cirrhosis

Several, predominantly retrospective, surveys have reported the spontaneous resolution of PVT, particularly for incomplete occlusions, in a percentage ranging from 0 to 70% of cases. Scant but interesting data suggest that the rate of spontaneous recanalization is higher in patients with Child–Pugh classes A and B and lower in patients with advanced liver failure, potentially as a consequence of low portal vein flow velocity or a stronger prothrombotic balance associated with advanced cirrhosis. Unfortunately, to date, no specific predictors of PVT regression have been identified. Although this issue remains controversial, current data suggest that, in the absence of intestinal ischemia (the only clear indication for treatment), the management of liver cirrhosis and its major complications should be maintained, regardless of changes in PVT [3,29,35].

In occlusive PVT or portal cavernoma, the probability of spontaneous resolution of PVT is low, independent of the choice to treat or not [1,3,35].
